# Diagnostic Accuracy of Echo-planar Diffusion-weighted Imaging in the Diagnosis of Intra-cerebral Abscess by Taking Histopathological Findings as the Gold Standard

**DOI:** 10.7759/cureus.4677

**Published:** 2019-05-16

**Authors:** Hina Siddiqui, Sameera Vakil, Maria Hassan

**Affiliations:** 1 Radiology, Dow University of Health Sciences (DUHS), Karachi, PAK; 2 Radiology, Dr. Ziauddin Hospital, Karachi, PAK

**Keywords:** intra-cerebral abscess, echo-planar diffusion-weighted imaging

## Abstract

Objective

To determine the diagnostic accuracy of echo-planar diffusion-weighted imaging (DWI) in the diagnosis of intra-cerebral abscesses by taking histopathological findings as the gold standard.

Subject and methods

A retrospective cross-sectional study was performed from July 2014 to June 2015 at a tertiary care hospital in Karachi. A total of 462 patients, who were referred for magnetic resonance imaging (MRI) brain, presenting with clinical suspicion of an intra-cerebral abscess on the basis of clinical signs and symptoms, were included in this study. MR imaging was performed. All patients subsequently underwent surgery. The histopathological findings of these patients were collected and compared with echo-planar diffusion-weighted MRI findings. A proforma was used to record the findings.

Results

The mean age of the patients was 47.39±13.54 years. The sensitivity, specificity, positive predictive value, negative predictive value, and diagnostic accuracy of echo-planar diffusion-weighted MRI in the diagnosis of intra-cerebral abscesses was 85.64%, 95.88%, 93.82%, 90.14%, and 91.56%, respectively.

Conclusion

Brain abscesses and necrotic tumors are, most of the time, difficult to differentiate on routine conventional imaging, and prompt diagnosis is important, as an untreated brain abscess could be lethal. Diffusion imaging can aid in the diagnosis and further management plan so as to help in improved patient care. Although this sequence has high sensitivity and specificity, it should be used in addition to conventional imaging and not as a replacement of histopathology.

## Introduction

An intra-cerebral abscess is a significant healthcare problem in the developing world due to large-scale poverty, illiteracy, and lack of hygiene [1-2]. It starts as a localized area of cerebritis, which is subsequently converted into a collection of pus within a well-vascularized capsule [2]. Predisposing conditions are present in up to 86% of patients and mostly consist of contiguous or distant foci of infection [3]. Intra-cerebral abscesses constitute up to 8% of the intracranial space-occupying lesions seen in the developing countries [4]. It is most common in the second to third decades of life, with a male to female ratio of 3.4:1 and a mortality rate of about 11.3% [5]. Its prevalence is anticipated to be 2%-14% [6].

An intra-cerebral infection is a true neurosurgical emergency that requires rapid diagnosis and appropriate management, as its clinical course may become fulminant [1]. Advances in surgery, neuroimaging, and antibiotics have significantly improved the outcomes, but mortality and morbidity remain high [1-2]. Computed tomography (CT) scans and magnetic resonance imaging (MRI) are the main noninvasive modalities used to diagnose intra-cerebral abscesses [4]. However, the imaging features of abscesses are nonspecific and may simulate those of cystic rim-enhancing tumors. Because of this limited ability of conventional CT and MRI, new and more advanced techniques are urgently needed [4,7].

Diffusion-weighted (DW) MRI using the echo-planar technique has been shown to be useful for distinguishing brain abscesses from necrotic or cystic brain tumors [8-9]. DW MRI characterizes a lesion based on the free diffusing property of water molecules in that lesion [10-11]. An intra-cerebral abscess causes diffusion restriction due to inflammatory cells, necrotic tissue, and proteins in the pus, which returns bright signals on DW MRI [4,10]. This feature differentiates it from necrotic tumors, which are usually not diffusion restricted [10]. This carries significant implications on the intent and nature of treatment [9]. Reddy et al. reported the sensitivity and specificity of echo-planar diffusion-weighted imaging (DWI) for detecting intra-cerebral abscesses as 96% and 96%, respectively [9].

This study aims to determine the diagnostic accuracy of echo-planar diffusion-weighted MRI in the diagnosis of intra-cerebral abscesses. If this study shows high sensitivity and specificity of echo planar DWI, then early diagnosis would eliminate the need for invasive diagnostic procedures, lead to early commencement of treatment, and, hence, will substantially reduce the morbidity of this condition. There are only a few studies on this topic in our population with relatively small sample size. In this study, we included 462 subjects, which gave stronger results.

## Materials and methods

A retrospective cross-sectional study performed for one year from July 2014 to June 2015 at a tertiary care hospital in Karachi. Non-probability, consecutive sampling was used. Informed consent was waived, as it was a retrospective study. A total of 462 patients of ages 18 to 70 yrs (mean age 47.39 ± 13.54 years), both genders, who were referred for MRI brain, presenting with clinical suspicion of an intra-cerebral abscess on the basis of clinical signs and symptoms, as described below:

- The clinical triad of moderate to severe headache (4-10 on the visual assessment scale), fever (>101 F), and altered mental status (assessed by Glasgow Coma Scale (GCS) ≤15) of one week or more in duration.

- Other associated features like generalized seizures (assessed clinically by jerky movements), neck stiffness, blurring of vision, vomiting (at least two episodes per day for at least one day), and limb weakness assessed by clinical examination.

The exclusion criteria included:

- Patients who do not undergo surgery or are lost to follow-up

- Previously diagnosed cases of an intra-cerebral abscess or brain tumor

- Patients in whom no ring-enhancing lesion is seen on brain MRI

- Previous history of radiotherapy, chemotherapy, or surgery

MR imaging was performed on a 1.5 Tesla system (Atlas, Toshiba, Japan). The protocol consists of T1-weighted spin-echo (SE) images (TR/TE: 615/12 ms), T2-weighted turbo SE (TR/TE: 4000/98 ms), fluid-attenuated inversion-recovery sequences (TR/TE: 9000/119 ms), and post-contrast T1- weighted images. Diffusion-weighted imaging was obtained in the axial plane using an echo-planar single-shot sequence with the following parameters: TR/TE 4500/83 ms; matrix size, 256× 256; FOV, 220 mm; slice thickness, 5 mm. The MRI was interpreted by a senior radiologist having more than seven years of experience.

All patients subsequently underwent surgery. The histopathological findings of these patients were collected and compared with the echo-planar diffusion-weighted MRI findings. A proforma was used to record the findings.

Statistical data analysis was done using the Statistical Package for the Social Sciences (SPSS version 20; IBM Corporation, Armonk, NY, US). The age and size of the abscess were presented by the mean and standard deviation. Frequency and percentage were computed for gender. A 2x2 table was constructed and the sensitivity, specificity, positive predictive value (PPV), negative predictive value (NPV), and diagnostic accuracy of echo-planar diffusion-weighted MRI for the diagnosis of an intra-cerebral abscess were calculated by taking histopathological findings as the gold standard. Kappa statistics were calculated to see the strength of the association between the two modalities. K >0.8 was taken as significant. Stratification with respect to the age and size of the abscess was done. Post-stratification kappa statistics were also calculated.

## Results

**Figure 1:**
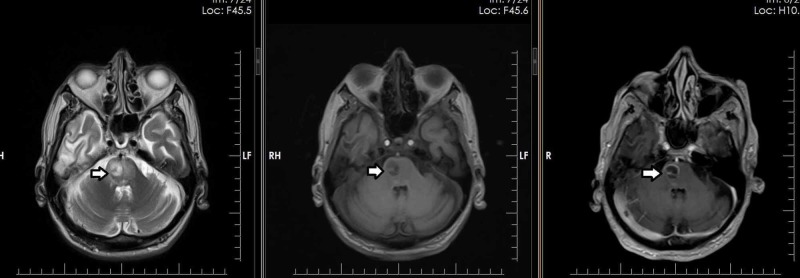
Seventy-year-old patient with a right hemipons abscess showing peripheral enhancement on post-contrast images

**Figure 2:**
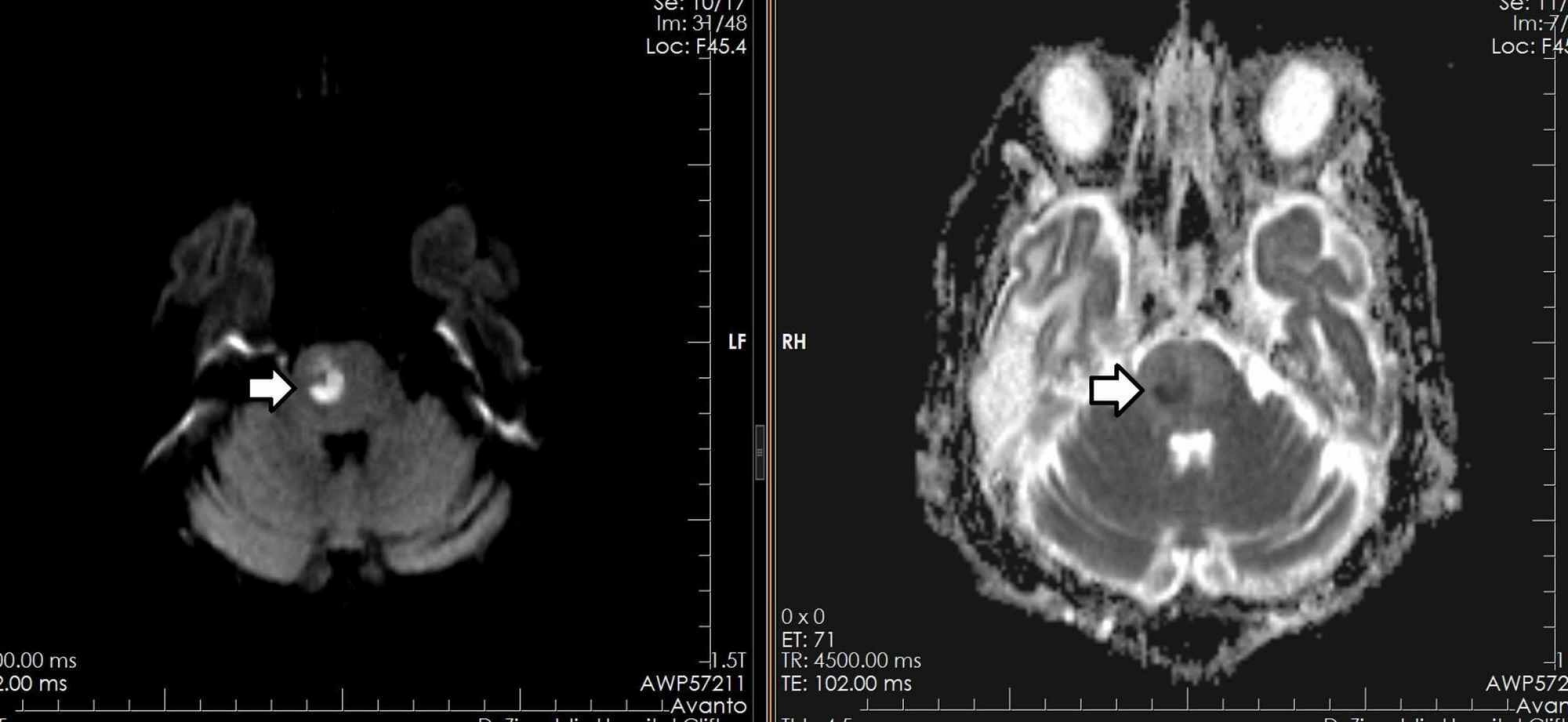
Similar patient with a right hemipons abscess showing diffusion restriction on DWI and ADC sequences DWI: diffusion-weighted magnetic resonance imaging; ADC: apparent diffusion coefficient

A total of 462 patients, who were referred for MRI brain, presenting with clinical suspicion of an intra-cerebral abscess on the basis of clinical signs and symptoms, were included in this study. One of the patients, age 70 years, showed a well-defined abnormal signal intensity area in the right hemipons on T2W and T1W images, with diffusion restriction and peripheral post-contrast enhancement suggestive of an abscess (Figures 1-2).

**Figure 3:**
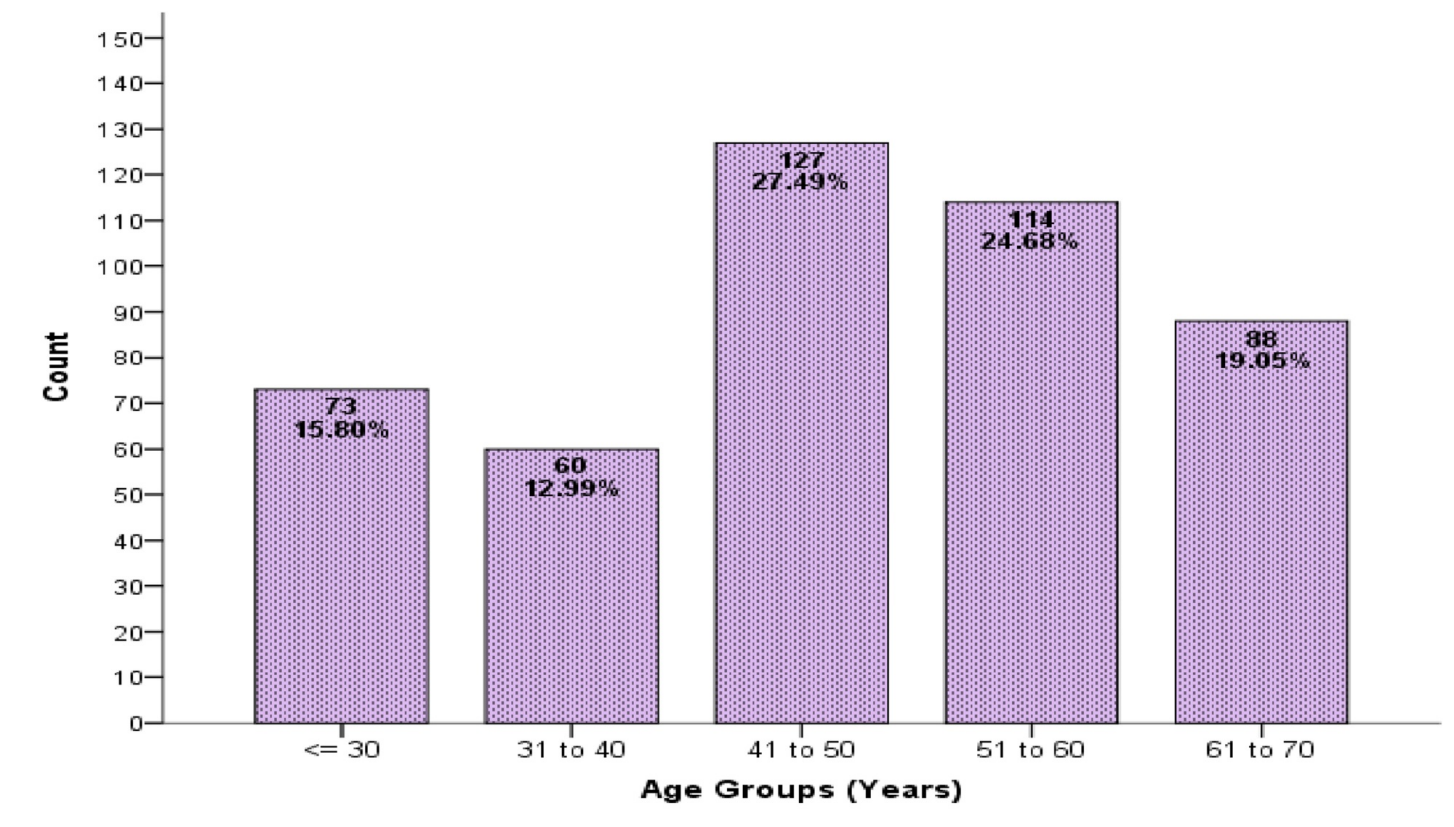
Age distribution of patients n=462 patients

Regarding the demographics of the patient, it was observed that patients above the age of 40 years were more affected (Figure 3).

The mean age of the patients was 47.39 ± 13.54 years; similarly, the mean size of the abscesses was 4.99 ± 2.26 cm. There were 58.66% male and 41.34% female.

Histopathology confirmed the diagnosis of an intra-cerebral abscess in 42.2% (195/462) cases while echo-planar diffusion-weighted MRI findings showed only 38.5% (178/462) cases had an intra-cerebral abscess. Regarding the false echo-planar diffusion-weighted MRI, 11 of 178 cases were false positive and 28 of 284 were diagnosed as false negative finding by echo-planar diffusion-weighted MRI. The sensitivity, specificity, PPV, NPV, and diagnostic accuracy of echo-planar diffusion-weighted MRI in the diagnosis of intra-cerebral abscesses was 85.64%, 95.88%, 93.82%, 90.14%, and 91.56%, respectively. Kappa statistics (k=0.83) also showed excellent agreement between findings. A 95% confidence interval was also reported for each statistics (Table 1).

**Table 1:** Diagnostic accuracy of echo-planar diffusion-weighted imaging in the diagnosis of intra-cerebral abscesses by taking histopathological findings as the gold standard

Parameter	Estimate	Lower-Upper (95% CIs)
Sensitivity	85.64%	(80.03, 89.88 )
Specificity	95.88%	(92.77, 97.68 )
Positive Predictive Value	93.82%	(89.27, 96.51 )
Negative Predictive Value	90.14%	(86.12, 93.09 )
Diagnostic Accuracy	91.56%	(88.67, 93.76 )
Kappa Value	0.83	(0.734 - 0.91)

A stratification analysis was performed and observed that the diagnostic accuracy of echo-planar diffusion-weighted MRI in the diagnosis of intra-cerebral abscesses and Kappa statistics was high in all age groups and both sizes of abscesses (≤5 cm, >5 cm) (Tables 2-5).

**Table 2:** Diagnostic accuracy of echo-planar diffusion-weighted imaging in the diagnosis of an intra-cerebral abscess for below and equal to 50 years of age

Parameter	Estimate	Lower-Upper (95% CIs)
Sensitivity	90.98%	(84.57, 94.89)
Specificity	97.1%	(92.78, 98.87)
Positive Predictive Value	96.52%	(91.4, 98.64)
Negative Predictive Value	92.41%	(86.93, 95.71)
Diagnostic Accuracy	94.23%	(90.7, 96.47)
Kappa Value	0.88	(0.76 - 1.0)

**Table 3:** Diagnostic accuracy of echo-planar diffusion-weighted imaging in the diagnosis of an intra-cerebral abscess for above 50 years of age

Parameter	Estimate	Lower-Upper (95% CIs)
Sensitivity	76.71%	(65.83, 84.92 )
Specificity	94.57%	(89.22, 97.35)
Positive Predictive Value	88.89%	(78.8, 94.51)
Negative Predictive Value	87.77%	(81.29, 92.22)
Diagnostic Accuracy	88.12%	(82.93, 91.88)
Kappa Value	0.7347	(0.59-0.87)

**Table 4:** Diagnostic accuracy of echo-planar diffusion-weighted imaging in the diagnosis of intra-cerebral abscesses that are ≤5 cm in size

Parameter	Estimate	Lower-Upper (95% CIs)
Sensitivity	81.36%	(73.38, 87.35)
Specificity	97.35%	(93.39, 98.97)
Positive Predictive Value	96%	(90.16, 98.43)
Negative Predictive Value	86.98%	(81.08, 91.24)
Diagnostic Accuracy	90.33%	(86.21, 93.32)
Kappa Value	0.80	(0.68 - 0.91)

**Table 5:** Diagnostic accuracy of echo-planar diffusion-weighted imaging in the diagnosis of intra-cerebral abscesses that are >5 cm in size

Parameter	Estimate	Lower-Upper (95% CIs)
Sensitivity	92.21%	(84.02, 96.38)
Specificity	93.97%	(88.07, 97.05)
Positive Predictive Value	91.03%	(82.62, 95.58)
Negative Predictive Value	94.78%	(89.08, 97.59)
Diagnostic Accuracy	93.26%	(88.82, 96.02)
Kappa Value	0.85	(0.71 - 1.0)

## Discussion

A brain abscess remains a diagnostic challenge to clinicians and radiologists because the presenting clinical manifestations and neuroradiologic appearances are often nonspecific. Only 40%-50% of patients are febrile on examination. The more common signs and symptoms are those of any expanding intracranial mass: headache, altered mental status, focal sensorimotor deficits, seizure, nausea, and vomiting [10]. For this reason, a radiological diagnosis has particular importance [11]. Fever is characteristic of the invasion period of an abscess, whereas its encapsulation results in the normalization of body temperature; hence, the diagnosis depends on radiological investigations [11]. Sometimes, CT and conventional MRI cannot easily differentiate cystic and necrotic brain tumors from abscesses. Ebisu et al. were the first to describe the role of DWI here. The authors reported that abscesses appeared hyperintense on DWI and hypointense on apparent diffusion coefficient (ADC) mapping, whereas tumors appeared hypointense on DWI and hyperintense on ADC mapping [12]. Diffusion-weighted imaging is more practical in clinical use. It is a fast echo planar imaging technique and requires less imaging time [13]. CT and MR imaging has high sensitivity for diagnosing ring-enhancing brain lesions but most of the time, it is difficult to differentiate between a neoplastic mass lesion and a brain abscess. DWI shows much promise in differentiating the differentials of ring-enhancing lesions [14].

A total of 462 patients, who were referred for MRI brain, presenting with a clinical suspicion of an intra-cerebral abscess on the basis of clinical signs and symptoms, were included. There were 58.66% male and 41.34% female in our study; this is in agreement with most series where males are affected more frequently than females [15-16]. We found above 40 years of age were more reflected and the mean age of the patients was 47.39±13.54 years. Roche et al. in 2003 and Kastenbauer et al. in 2004 reported that a brain abscess is most common in the third decade of life but may occur at any age [16-17]. Abscesses due to para-nasal infections are most common between the ages of 10 and 30 years. Otogenic abscesses are most common in childhood and after 40 years of age.

Previous studies described a high accuracy of DWI in differentiating brain abscess from cystic/necrotic tumors. The differentials of ring-enhancing lesions include high-grade gliomas, metastases, abscesses, demyelinating diseases, and resolving hematomas [18]. There are reports that diffusion restriction can be seen in neoplastic cystic lesions. Similarly, increased diffusion and ADC value have been described, thus raising the question mark over the accuracy of this technique [19-20]. The sensitivity, specificity, PPV, NPV, and diagnostic accuracy of echo-planar diffusion-weighted MRI in the diagnosis of intra-cerebral abscesses was 85.64%, 95.88%, 93.82%, 90.14%, and 91.56%, respectively. Kappa statistics (k=0.83) also showed excellent agreement between findings. NInety-five percent confidence was also reported for each statistic. This study showed high sensitivity, specificity, positive, negative predictive value, and accuracy, which is in agreement with other studies. Lai et al. reported that DWI yielded a sensitivity of 93.33%, a specificity of 90.91%, a PPV of 93.33%, and an NPV of 90.91% [21], and Reddy et al. reported the sensitivity of DWI for the differentiation of brain abscesses from non-abscesses was 96%; specificity, 96%; positive predictive value, 98%; negative predictive value, 92%; and accuracy was 96% [9].

## Conclusions

Brain abscesses and necrotic tumors are, most of the time, difficult to differentiate on routine conventional imaging, and prompt diagnosis is important, as an untreated brain abscess could be lethal. Diffusion imaging can aid in the diagnosis and further management plan so as to help in improved patient care. Although this sequence has high sensitivity and specificity, it should be used in addition to conventional imaging and not as a replacement for histopathology.
